# Multiobjective dynamic resource allocation in cloud computing using Harris Hawk Optimization Algorithm (MDLB-HHO)

**DOI:** 10.1371/journal.pone.0351653

**Published:** 2026-06-25

**Authors:** Varun C. M, Anto Kumar R. P, Paulraj D, Priyanka P. S

**Affiliations:** 1 Department of Computer Science and Business Systems, R.M.K. Engineering College, Kavaraipettai, Tamil Nadu, India; 2 Department of Computer Science and Engineering, St. Xavier’s Catholic College of Engineering, Chunkankadai, Tamil Nadu, India; 3 Department of Computer Science and Engineering, R.M.K. Engineering College, Chennai, India; 4 Department of Computer Science and Engineering, R.M.K. College of Engineering and Technology, Chennai, India; Dr Shakuntala Misra National Rehabilitation University, INDIA

## Abstract

To increase cloud computing utilization and performance, efficient load balancing and resource distribution techniques are essential. Dynamic load balancing and resource allocation in cloud systems is necessary due to a number of reasons, but this is not an easy and straightforward task. The primary goal of dynamic load balancing of cloud systems is to optimize the workload and resource utilization. The Harris Hawks Optimization (HHO) algorithm is a dynamic method of allocating the workloads to the virtual machines (VMs) according to the workload distribution and the use of the resources. The comparison of experimental analysis and other load-balancing methods shows that the HHO algorithm can be used to control dynamic load balancing in a rather efficient and effective way. With such technical developments, there has been a decrease in time taken to respond as well as the use of resources. The suggested solution is a cost-effective and efficient solution to the load-balancing problem in dynamic conditions and is based on the collaborative hawks hunting behavior. The system converts the resource allocation scheme to the changeable cloud application requirements. This is achieved by a multiobjective fitness function which aims to maximize the efficiency of resources, minimize the response time and resource usage. The primary objective of the study is to ensure that the clouds services become effective and sustainable. The Harris Hawks discover the most optimal distribution techniques of activities by closely observing the space of solutions. They then apply positional updates and iterative interactions to adapt to changing workloads. The system dynamically assigns jobs to virtual machines (VMs) without compromising load balance and efficient resource use through the use of the cooperative search behavior of the hawks. The proposed solution effectively manages the cases when the task requirements are constantly changing. Applying a multiobjective fitness function greatly improves key performance metrics like overall performance, resource usage, and reaction time. This study demonstrates how the HHO algorithm increases the effectiveness and robustness of cloud-based services in dynamic operational environments.

## Introduction

The more cloud-based services and applications there are, the more significant load balancing and effective resource allocation are. Resource management and efficient load distribution are some of the most crucial elements in enhanced performance and use of available resources as much as possible. Through the dynamic distribution of tasks over several virtual machines, load balancing solutions improve response time and maximize resource efficiency. Historically, load balancing strategies have prioritized either optimizing resource utilization or reducing response time. However, load balancing is a multi-objective problem that necessitates weighing trade-offs between conflicting objectives in real-world cloud computing infrastructures. As an example, the optimization of resource usage can increase response time, and vice versa. Thus, load-balancing strategies are required that can effectively deal with the multiobjective character of the problem.

Even though resource allocation and load balancing are essential elements of cloud computing, the efficacy of current methods and approaches is frequently lacking. Inspired by biological and natural processes, meta-heuristic algorithms have demonstrated great promise in solving challenging optimization problems in this field. HHO technique has proved to be very effective in many optimization issues. It is founded on collaborative hunting strategies of Harris hawks. Cloud computing is a viable method for multi-objective load balancing and resource allocation because it offers a good mix between investigation and exploitation. Such efficient methods are becoming more and more necessary as cloud environments grow to handle more users and resources, which increases system complexity.

Conventional methods for resource allocation and load balancing frequently struggle to keep up with the increasing complexity. At higher levels, it can be very difficult to ensure the long-term effectiveness of these strategies. The cloud infrastructures normally consist of numerous resources of varying capabilities. The efficiency of load balancing and resource allocation may be impacted by their limited capacity to use heterogeneous resources effectively. Furthermore, most cloud-based apps have workloads that are erratic and prone to variations. Thus, the latency of load balancing and resource allocation algorithms can be low to changes of this kind and this will result in poor performance. The resource fragmentation effect is also likely to have an impact on the multi-tenant cloud system where resources are divided into small units, which are either inefficient or not convenient to utilize and, therefore, reduce the overall resource accessibility. A significant limitation of dealing with geographically dispersed resources also exists. Load balancers and resource allocation algorithms are not fault tolerant and highly available in some cases, which results in a service outage due to hardware or software failures.

The article presents a new application of the Harris Hawks Optimization (HHO) algorithm to the resolution of the resource allocation and multi-objective dynamic load balancing issues in cloud computing. The proposed method aims to ensure that the distribution of work is both fair and efficient by maximizing such significant variables as cost, energy efficiency, resource consumption, and response time. The inclusion of the multi-objective nature of the problem into the HHO framework offers a comprehensive and reliable optimization solution. It dynamically allocates jobs to the available virtual machines (VMs) using patterns of workload and resource consumption [1]. In this fashion, it will be imitating the collaborative hunting behavior of the Harris hawks which have their hierarchical position of exploratory, sentinel and possessive. By carefully examining the solution space and assessing the efficacy of suggested solutions, the hawks collaborate to enhance load balancing and resource allocation.

The proposed solution is also rigorously evaluated with the help of a complete test suite and contrasted with the current cloud computing methods of load balancing and resource distribution [2]. The essential performance parameters such as response time, cost, energy consumption, and resource use are studied in detail. The findings indicate that the multiobjective meta-heuristic approach, comprising the Harris Hawk Optimization (HHO) algorithm, is superior to the traditional methods of successfully solving the dynamic load balancing and resource allocation challenges. This method is an effective way in which a tradeoff between conflicting goals is sought and overall performance of the system is enhanced.

This paper is aimed at using the Harris Hawk Optimization (HHO) algorithm to determine the most effective cloud resources allocation and multiobjective dynamic load balancing strategies. The plan is highly concerned with effective resource use, flexibility to the changing workload and provision of high availability and fault tolerance to handle the growing complexity of cloud computing environments [3].

The proposed system is called HHO algorithm and is founded on the collaborative hunting of Harris hawks. It is an active strategy regarding the division and assignment of the resources to the virtual machines in regards to workload and resource consumption. Optimization plan is implemented based on several criteria, which include response time, resource utilization, energy efficiency, and cost. It has been more successful than other multi-objective metaheuristic approaches in the experimental tests carried out in the cloud computing environments. The strategy is way better than the traditional load balancing and resource allocation strategies since it enhances the performance of an entire system and it also addresses the conflicting objectives. The primary contribution of the study is the development of a viable multi-objective model of resource management and dynamic load balancing intended to enhance the effectiveness and overall performance of cloud-based services.

### Main Contribution of the research

- The article presents the MDLB-HHO for cloud computing using the Harris Hawk Optimization (HHO) method.
- The proposed method dynamically assigns tasks to virtual machines by simulating the cooperative foraging behavior of hawks. It incorporates a multiobjective fitness function to optimize key metrics by minimizing response time and resource overuse while maximizing efficiency.
- According to experimental results, MDLB-HHO outperforms existing techniques in handling dynamic workloads, enhancing the sustainability, responsiveness, and resource utilization of cloud services.

### Organization of the research

The rest of the paper is structured as follows: A thorough literature overview of cloud computing load balancing, resource allocation, and meta-heuristic techniques is given in Section 2. The technique, including the formulation of the problem, specifics of the HHO algorithm, and the incorporation of multi-objective optimization, is presented in Section 3. Section 4 provides a detailed analysis, results, and a description of the experimental setting. The findings are summarized, the research’s contributions are highlighted, and future directions for enhancing multi-objective dynamic load balancing and resource allocation in cloud computing utilizing the HHO algorithm are outlined in Section 5.

## Literature review

In cloud computing, load balancing and resource allocation are essential because they guarantee optimal resource use, encourage equitable work distribution, and greatly improve system performance. Meta-heuristic algorithms that draw inspiration from biological or natural processes have demonstrated significant promise in tackling these problems. Harris Hawk Optimization (HHO) is one such method that simulates the cooperative hunting style of Harris hawks. Although HHO offers a viable way to handle challenging optimization tasks, its full promise in cloud environments particularly for load balancing and resource management remains unrealized.

The meta-heuristic methods for load balancing and resource allocation in cloud computing systems are the primary focus of this literature review, with special attention paid to Genetic Algorithms, Particle Swarm Optimization, Ant Colony Optimization, and Simulated Annealing. It looks into the application of different methods of allocating resources and managing demand efficiently and also the advantages and shortcomings of each method. D. A. Shafiq and others suggested a data center load-balancing system to maintain the same level of performance in cloud computing. Their plan involved the use of multiobjective optimization to realize an effective load balancing within data centers. By analyzing how changes in parameters affect the algorithm’s performance, the sensitivity study was carried out to confirm its efficacy. The algorithm’s adaptability and durability under various workload situations were highlighted in the study. The article successfully touches on significant concerns that revolve around job distribution and resource allocation by providing a new solution to workload balance in cloud computing, which can be utilized to develop better load-balancing algorithms in cloud systems.

The study [4] critically analyzes the shortcomings of current load-balancing techniques in big data cloud systems and highlights the significance of effective load distribution in cloud computing. To overcome these obstacles, the Central-Regional Architecture-Based Load Balancing Technique (CRLBT) is suggested by the authors. They combine two optimization algorithms Throughput Maximized Particle Swarm Optimization (TM-PSO) and Throughput Maximized Firefly Optimization (TM-Firefly) within a specific throughput maximization framework to improve load-balancing efficiency. An amalgamation of these methods offers CRLBT a significant leap forward in comparison to the traditional hierarchical, distributed and centralized cloud systems. Experimental evidence indicates that CRLBT is a good load balancing optimizer in big data cloud system because the results indicate that there was a great improvement in the CPU utilization, response time, task rejection rate and network throughput.

The study [5] states that fog computing is becoming more widely acknowledged as an essential strategy for controlling data flow in expansive and intricate Internet of Things (IoT) networks. The Dynamic Energy-Efficient Resource (DEER) technique is presented in the paper to facilitate effective load balancing in such fog conditions. This strategy is intended to lower the amount of energy consumption, emission of carbon and operating costs and enhance performance of systems. The Tasks Manager, Resource Engine, Resource Information Provider, Resource Scheduler, Resource Load Manager, and Resource Power Manager are the six main parts of the DEER architecture. Task submission is managed by the Tasks Manager, and pertinent resource data is provided by Cloud Data Centers. The Resource Scheduler dynamically distributes resources according to current demand, while the Resource Engine assigns tasks to the proper resources. In an effort to maximize energy use, the Resource Load Manager and Resource Power Manager constantly assess the condition of the resources. Such an approach will maximize energy use and compute costs, thereby, maximizing performance and minimizing environmental impacts of fog-based systems.

This study [6] provides a new approach that combines the Modified Harris Hawks Optimization (MHHO) algorithm with a layer-fitting strategy to address work scheduling and resource allocation issues in fog computing scenarios. The major goal is to reduce operational expenses and maximize resource usage by implementing effective load balancing between fog and cloud levels. Given the large data quantities created by the Internet of Things (IoT), efficient job distribution is critical but still a difficult challenge. The layer-fitting method maintains this to the minimum by not overloading the resources, exhausting the resources and compromising the services through the even distribution of workloads across the different layers based on the priority. The Modified Harris Hawks Optimization (MHHO) method assigns jobs dynamically to the most relevant resources at all levels. The suggested approach attempts to enhance overall efficiency by reducing makespan, execution costs, and energy usage. Its performance will be compared to classic optimization methods such as the Firefly Algorithm (FA), Ant Colony Optimization (ACO), Particle Swarm Optimization (PSO), and standard HHO, showing MHHO’s effectiveness when combined with the layer-fitting algorithm.

Cloud computing makes it difficult to manage enormous volumes of data, and even a single connected virtual machine (VM) failure can cause serious system disruptions. To counter this, Edward et al. [7] point out the importance of effective load balancing. To enhance VM management and system stability, they propose a new resource allocation scheme in their paper that combines transfer learning and the Fruitfly Optimization Algorithm. The first phase entails assigning various user tasks to the virtual machines; the second phase is the allocation of the load to all the virtual machines connected so that balance is achieved. The execution of the work is done in priority manner and resources are shared. The study ends with the conclusion that, due to the effective solution of the problem of resource distribution and workload planning, the Fruitfly-based transfer learning method significantly enhances load balancing in cloud computing. It therefore leads to improved performance of the system and improved quality of the cloud services.

Praveenchandar and Tamilarasi [8] introduced a novel load-balancing strategy, PBMM, to increase dynamic resource allocation in cloud computing systems. To make the system more stable and profitable, this strategy is aimed at efficient task planning and resource distribution as well as addressing the load balancing problems directly. The algorithm considers important factors such as the size of work and value of customer bid. In [9], a related work employs the use of task and resource tables to minimize the average waiting and response times of the users. System stability can be even improved and revenue can be raised by involving more high-priority users. As per simulation data, the proposed solution is able to achieve its objectives by distributing resources and tasks in the most productive way, generating more revenue and making the system more stable in the process. The recommended algorithm considers the size of the tasks, the cost of the bid and utilizes resource and task tables hence stable, less waiting and more profitable. The findings show that load-balancing strategies are mandatory in ensuring efficient utilization of resources and quality service provision, which promotes the adoption of cloud computing strategies.

Y. Sun et al. [10] have resolved the problem of physical memory deficiency, underutilization of resources and profitability of ASP in MEC environment in detail. Their ASP profit-conscious strategy can be also extended to optimize the resource utilization, the latency and the long-term profitability in terms of Lyapunov optimization framework and genetic algorithms. A stochastic optimization task with long-term ASP profit restrictions is how the problem is stated. To address this problem, the authors reformulate it as a time-slot based optimization problem that belongs to the paradigm of Lyapunov optimization. Then they employ Genetic Algorithms (GA) to develop an online heuristic algorithm that produces nearly optimal tactics in each time slot. Another solution is the one that was proposed by Chetan Kumar et al. [11], and it is focused on minimizing the system latency and maximizing the ASP profitability. Their remedy is a good use of the available edge network resources since it is the most effective method of loading applications, task assignment, and distribution of compute resources concurrently. Not only does this reduce the long-term latency, but also increases the number of ASVs since they will be able to reach the desired profitability.

Devi et al. [12] proposed a security model of deep learning to enhance job scheduling and effectively manage security concerns in cloud computing. The study proves that deep learning is better than the conventional scheduling techniques and the benefits of using it in the safe execution of activities. The ability of the model to address security concerns effectively in the scheduling process is an indicator that it is important to design strong security processes in the cloud systems. The results indicate that the model is superior to the traditional approaches and security-based strategies ought to be implemented in the cloud.

According to Abhikriti and Sunitha [13], scheduling algorithms are essential to cloud computing as they increase resource utilization and decrease Makespan time. Nonetheless, the majority of the modern algorithms tend to minimise Makespan, which leads to load imbalance and inefficient resource usage. The proposed study introduces a new method of addressing these problems by applying the Credit-Based Resource-Aware Load Balancing Scheduling Algorithm (CB-RALB-SA). By matching task workloads to the processing capabilities and current load levels of the available resources, this technique guarantees equitable job distribution. It has a credit based scheduling scheme in which weights are allocated during task to resource mapping. The mapping takes advantage of the Resource Aware and Load (RAL) approach that deploys the Honey Bee Optimization heuristic and FILL and SPILL functions to enhance performance.

Reference [14] highlights the major issues of load balancing in cloud computing, highlighting the pressing need to address this issue. In addition to evaluating several optimization approaches, such as Genetic Algorithms, Ant Colony Optimization (ACO), Particle Swarm Optimization (PSO), the BAT algorithm, and Grey Wolf Optimization (GWO), the study investigates Swarm Intelligence (SI) as a possible methodological framework. Because of their efficacy, PSO and GWO are given special consideration among these. To ensure the safe and efficient use of data across platforms, the concept of federated clouds, which is the combination of both the private and the public cloud environments, is also discussed. Federated clouds, however, may be tricky when it comes to authorization and authentication. A very recently proposed technique is the Secured Storage and Retrieval technique (ATDSRA) which has been postulated to allow the users of the both the private and the public cloud databases to store and retrieve their information in a secure manner [15]. It uses data aggregation and merge methods, and the Triple-DES encryption to render it more secure. Further, to support access control on the federated cloud data and to ease the auditing process, CRT-based Dynamic Data Auditing Algorithm (CRTDDA) is proposed.

The security of federated data kept in cloud settings is greatly improved by the ATDSRA method in conjunction with the CRTDDA auditing scheme. The suggested approach ensures safe data storage, retrieval, and auditing while successfully addressing the intricate problems of cloud authorization and authentication. The authors in [16] present a hybrid strategy that combines the Firefly and BAT algorithms to address the load balancing problem. This hybrid approach maximizes resource allocation and enhances system performance by utilizing the Firefly algorithm’s global optimization capabilities and the BAT algorithm’s quick convergence. The study indicates that substantial improvements can be made as in the reduction of the total response time, and the globally optimized, fast convergence. Task scheduling has a major impact on resource usage and cloud computing system performance as a whole, claim Saydul et al. [17]. They emphasize how effective job scheduling strategies are necessary to prevent resource waste and performance deterioration. Their study looks at the advantages and disadvantages of different scheduling techniques used in grid and cloud computing settings, as well as the difficulties that come with them. To address the gaps in the already existing body of research and enable the development of more effective scheduling techniques, the authors create a taxonomy that allows classifying and comparing different techniques in a systematic fashion.

To accomplish equitable job distribution, Junaid et al. [18] suggest a priority-based job scheduling approach for cloud computing platforms. Enhancing overall system performance and optimizing resource consumption are the main goals of this strategy. The paper determines several issues that need to be investigated and which can lead to the development of superior scheduling techniques. Academicians, policymakers and practitioners can employ the priority-based scheduling method to optimize cloud computing settings since it enables formulation of more effective task scheduling plans. The problem of resource distribution in cloud computing, particularly in large-scale distributed systems, was the main emphasis of Ashawa et al.‘s study [19]. The principal objective is to assign resources optimally to maximize the overall computing performance or throughput. The paper also recognizes the disparities that exist between cloud and grid computing as well as the challenges that come with the distribution of the virtualized resources in a cloud computing platform.

The dynamic resource allocation system used in this work optimizes real-time resource distribution according to usage patterns by utilizing the Long Short-Term Memory (LSTM) technique. Simulations that closely resemble real-world situations are used to train and assess the LSTM model. The study also looks at how dynamic routing techniques might be incorporated into cloud data centers to improve traffic management. Compared to alternative load-balancing strategies, the proposed resource allocation model [20] demonstrates higher accuracy rates and lower error percentages in average request blockage probability under high traffic loads. Additionally, it improves network utilization while reducing processing time and resource consumption. In order to accomplish effective load balancing in energy cloud systems, future research should investigate the integration of various heuristic techniques with machine learning approaches, with a focus on the use of firefly algorithms.

Despite the fact that cloud computing offers consumers a wide selection of services, system breakdowns are becoming more likely due to growing demand. Numerous fault tolerance strategies have been developed to solve these issues and lessen their impact. The research [21] outlines a multilevel fault tolerance system that would provide better availability and reliability of cloud environments. The offered system works on two levels. On the first level, an algorithm of reliability assessment is used to determine reliable virtual devices. This will minimize the chances of error because it will analyze the reliability of virtual machines and allocate tasks to the most reliable. The second level is where a replication technology is used to ensure that data is available all the time through replication and dissemination of information on several physical or virtual computers. The technique will make sure that as long as one copy is not accessible or fails, there will still be other copies hence data availability. This multilevel fault tolerance strategy integrates the application of reliable virtual machine identification with data replication to offer fault resilience in real-time cloud environments resulting in fewer errors and the increased efficiency of the cloud services.

The issue of effective load balancing in dynamic and distributed systems is argued by Mishra and Manjula [22]. The study suggests that Multiobjective Memetic method (MOMA) should be applied to this problem. This algorithm uses local search method to solve convergence issues and concentrate on efficient job assignment to the available resources. It has also been indicated in the article that the Adaptive Plant Intelligent Behavior Optimization (APIBO) technique is used to re-schedule jobs in a manner that enhances fault tolerance. Moreover, a novel hybrid method called Multiobjective Whale Optimization-based Differential Evolution (M-WODE) is suggested in paper [23]. It is a hybrid of the Whale Optimization approach (WOA) and the Differential Evolution (DE) algorithm. This hybrid exploits the fast convergence and the bubble-net hunting mechanism of WOA, and the local search ability and exploration of the solution space are enhanced with the help of DE. DE is applied to optimise the Pareto front generated by the algorithm to prevent WOA being trapped in local optima.

The issues of efficient resource allocation in the modern cloud computing data centers where the resources are allocated and de-allocated dynamically have been discussed in the previous literature [24]. This situation increases the significance of effective virtual machine (VM) scheduling. This paper applies the Improved Water Optimization approach (IWOA) to propose a novel approach to Virtual Machine (VM) allocation in Infrastructure-as-a-Service (IaaS) cloud systems. The method is meant to address the NP-hard scheduling issue that such systems frequently find themselves in. The proposed approach is more efficient than other approaches such as Genetic Algorithms (GA), NSGA-III, and Particle Swarm Optimization (PSO) in terms of time and cost-effectiveness according to the results of experiments.

The authors of [25] propose a distinct approach to resource allocation in cloud computing by considering the changing user and application requirements. The approach improves the process of identifying and exploiting optimization opportunities by incorporating some factors in an Adaptive Multiobjective Teaching-Learning Based Optimization (AMO-TLBO) algorithm. These are availability of teachers, course materials flexibility, provision of structured and unstructured study time and provision of self directed research. Besides, the non-dominated solutions are evaluated on a grid-based system in an external repository. The AMO-TLBO framework is geared towards the balance of the load among the virtual machines and the optimization of the resource utilization in an effort to maximize on time and cost efficiency. The methodology is superior in all the categories which are evaluated and it performs better than the state-of-the-art algorithms like TLBO, MOPSO and NSGA-II.

The authors have introduced a distinct method, Multiobjective Particle Swarm Optimization with Crowding Distance (MOPSO-CD) to improve service composition in cloud-based Internet of Things (IoT) scenarios [26]. The approach utilizes Crowding Distance along with MOPSO to maximize more than one objective. MATLAB was used to test the strategy and compare its performance with three multi-objective algorithms that are already in use. The experimental results show that the MOPSO-CD algorithm provides a lower energy consumption rate and is more efficient than the strategies that are compared in terms of availability, reliability, response time, and latency.

Resource discovery protocol uses agents to broadcast messages of client queries to what is referred to as representative peers that contains information of the resource [27]. Even without a comprehensive understanding of the global state of the system, the agents and messages use marker-based stigmergy to self-organize. Simulation studies are carried out to assess how well the resource mapping (ARMAP) and resource discovery (ARDIP) protocols organize resources and shorten user search times.

In computational grids, a self-organizing and decentralized peer-to-peer (P2P) information system may be constructed based on a bio-inspired Antares algorithm [28]. Antares is based on ant colonies where swarm intelligence is a result of simple tasks done by individual agents at local level. The agents make copies and arrange descriptions on local grid hosts by peer-to-peer links to cluster similar ones. The system is self-adaptive to grid conditions and utilizes self-organizing nature of ant-like agents (Table 1).

**Table 1:** Comparison of existing methods.

Citation	Methodology	Advantage	Limitations
Ali Asghar Heidari et al., 2019	Meta-heuristic algorithms (e.g., HHO)	Effective optimization capabilities, near-optimal solutions	HHO implementation in cloud computing is largely unexplored
K Vinoth Kumar & A Rajesh, 2022	Multiobjective optimization strategy	Load balancing method for data centers, efficient algorithm, adjustable to different workload circumstances	Limited discussion on the algorithm’s resilience and adaptability
Shafiq D A et al., 2021	CRLBT strategy with TM-PSO and TM-Firefly	Improved response time, task rejection ratio, CPU utilization rate, and network throughput	**--**
Oduwole O A et al., 2022	DEER strategy	Increased efficacy, reduced energy consumption, decreased environmental impact	Limited discussion on the dynamic nature of fog environments
Rehman A U et al., 2020	Layer fit algorithm and MHHO strategy	Enhanced performance metrics, reduced costs, maximized resource utilization, load balancing in fog computing	Comparison with traditional optimization algorithms
Edward G, Geetha B & Ramaraj E, 2023	Fruitfly-based transfer learning	Improved load balancing, resource sharing, and task scheduling	Limited discussion on the algorithm’s performance compared to others
Praveenchandar & Tamilarasi, 2022	PBMM algorithm	Dynamic resource allocation, improved load balancing stability and profitability	**--**
Narwal A & Sunita D, 2023	CB-RALB-SA algorithm	Balanced distribution of tasks, efficient load balancing, honey bee optimization	**--**
Al Reshan M.S. et al., 2023	SI, PSO, and GWO algorithms	Potential load balancing solutions, comparison with other algorithms	**--**
Sermakanu A.M., 2020	ATDSRA and CRTDDA algorithms	Secure storage and retrieval, restricted data access	Limited discussion on the algorithm’s performance compared to others
Saydul A.M. et al., 2022	Task scheduling techniques analysis	Efficiency of job scheduling, resource utilization, performance improvements	Taxonomy proposed for classification and analysis of scheduling techniques
Ashawa M et al., 2022	LSTM-based dynamic resource allocation	Maximization of computing efficiency, improved resource allocation	Limited discussion on the comparison with other allocation techniques
Hariharan B, 2020	Resource allocation model	Increased accuracy, decreased error percentages, improved network utilization	Suggested further research on heuristics and machine learning techniques

### Summary of the literature review

The purpose of this study of the literature is to investigate the difficulties that cloud computing environments face when allocating resources and balancing loads. In order to tackle these problems, it also looks at and contrasts other approaches that researchers have suggested. Utilizing meta-heuristic methods, particularly the Harris Hawk Optimization (HHO) algorithm, to enhance load balancing and resource management is given significant attention.

A study investigates the use of popular meta-heuristic algorithms in cloud computing, namely in the areas of load balancing and resource allocation. These techniques include Genetic Algorithms, Particle Swarm Optimization, Ant Colony Optimization, and Simulated Annealing. It draws attention to each technique’s benefits, drawbacks, and working mechanisms. Another study uses a Central-Regional Architecture-Based Load Balancing Technique (CRLBT) to solve load distribution issues in large-scale cloud systems by introducing a multi-objective optimization strategy for data center load balancing. The development of DEER, a load balancing algorithm that is designed to successfully manage data flow in complex Internet of Things (IoT) environments, indicates the increased importance of fog computing. Moreover, another study recommends the application of layer-fit strategy alongside Modified Harris Hawks Optimization (MHHO) approach to optimize resource allocation and workload distribution in a fog computing environment. In a bid to make dynamic resource allocation in cloud computing setup more efficient, it also suggests a hybrid load balancing method that combines transfer learning and Fruit Fly Optimization Algorithm and a new method, called PBMM.

The research paper shows that security is a paramount aspect of the cloud computing scheduling because it presents a deep learning-based security framework and a Credit-Based Resource-Aware Load Balancing Scheduling algorithm, which are both designed to provide an efficient workload distribution. It suggests the Secured Storage and Retrieval Algorithm (ATDSRA) within a federated cloud and gives a deeper understanding of the use of the swarm intelligence techniques. LSTM-driven dynamic resource allocation, distributed resource management, cloud reliability evaluations, fault-tolerant architectures, task scheduling techniques, and priority-based scheduling algorithms are all included in the review. All things considered, it provides a thorough review of current developments in cloud computing load balancing and resource allocation, highlighting the increasing efficiency with which meta-heuristics and other optimization techniques may address these issues.

### Limitations

There is a need to conduct further research and develop load balancing and resource allocation strategies in cloud computing because there are gaps in the literature. Such research must aim at solving security issues, fitting into a particular environment, integrating hybrid optimization methods, and deploying dynamic resource allocation procedures. Also, the existing approaches should be reviewed, categorized, and evaluated to give directions and ideas to the development of more efficient solutions.

Only a few studies have been conducted on the use of the Harris Hawk Optimization (HHO) algorithm for resource allocation and load balancing in cloud computing.

Load balancing and resource allocation in cloud computing using meta-heuristic algorithms, HHO being one of them, is an untapped area as they have shown promise in solving optimization problems.

#### Lack of comprehensive analysis and comparison of meta-heuristic algorithms.

Load balancing strategies that are specific to certain cloud computing clouds like fog computing and big data clouds are necessary. The available studies tend to ignore the peculiarities of such settings and their difficulties.

Load balancing techniques peculiar to a particular cloud computing environment are not given enough attention.

Although load balancing and the process of resource allocation is very important in optimizing the performance of a system, care should be taken to ensure that security is also applied in the scheduling process. The security actions, such as deep learning-based security models, are to be incorporated into the environment of task scheduling, which should be the subject of further investigation.

#### Inadequate attention to security concerns in load balancing and resource allocation.

Process security scheduling is also important like load balancing and resource allocation in order to maximize the performance of the system. When it comes to job scheduling, the introduction of security measures, in particular, the ones that are based on deep learning should be researched.

#### Lack of emphasis on federated cloud environments.

The federated clouds which are a mixture of the public and the private clouds are a big challenge to safe data storage, authorization and authentication. One of the key research gaps is the insufficient models and algorithms that are able to address these complexities and improve the overall security of the federated cloud environments.

#### Limited exploration of combined optimization techniques.

More research is necessary to fully explore the possibilities of hybrid optimization algorithms, like the combination of Firefly and BAT algorithms, to enhance resource allocation and load balancing in cloud computing systems.

#### Incomplete understanding of the impact of scheduling on resource utilization and system performance.

It involves task scheduling to make the best use of resources and enhance the performance of the whole system. Further research needs to be conducted to develop more effective scheduling procedures that can balance the system load, make-span, and resource utilization with consideration of the performance trade-offs.

#### Inadequate investigation of dynamic resource allocation.

Techniques that are used in dynamic resource allocation must maximize computing efficiency, particularly in large-scale distributed computing systems. Further research on dynamic resource allocation models, like the ones based on LSTM algorithms, and their combination with dynamic routing strategies is required to control the traffic in cloud data centers.

### Motivation

The limitations of the existing system have motivated us to propose a novel method that performs better dynamically. The objective of this project is to generate a dynamic resource allocation system that can adapt to the changing requirements of cloud applications. Response time, efficiency, and resource usage are between the essential performance parameters that are optimized by the recommended technique using a multiobjective fitness function. In order to progress overall system performance and attain optimal resource usage in cloud environments, the study emphasizes the significance of flexible load balancing and resource management solutions.

## Objectives and problem statement

The main objective of the paper is as follows:

The main aim of cloud computing is to enhance the optimal utilisation of the available resources. This is done by reducing response time by the best allocation of workloads on several virtual machines (VMs) or central processing units (CPUs).

In order to underline the importance of the need to address the problems connected with the burden of the unexpected work, the approach of the load balancing may be considered as a potential solution as it may contribute to the distribution of the work based on the availability of the resources.

In order to accentuate the necessity to evaluate the ability of a resource to perform its duties and cover the demands of users, the optimal utilization of a resource with the help of its effective allocation turns out to be a desirable goal.

This study aims to examine existing load-balancing systems and assess their scalability and response time limitations. This highlights the need to prioritize the development of load-balancing technologies that are both more scalable and faster.

## Model development

Several virtual machines (VMs) manage work scheduling and load balancing in the cloud computing environment, as shown in Equation (1).


$$\begin{array}{c} \begin{array}{c} VM=\sum_{i=1}^{N}VM_{i}\\ Where\text{\textbackslash{}hspace\{0.17em}\;\}VM_{i}-i^{th}\text{\textbackslash{}hspace\{0.17em}\}Virtual\text{\textbackslash{}hspace\{0.17em}\}Machine\text{\textbackslash{}hspace\{0.17em}\} \end{array} \end{array}$$ (1)

In order to ensure that the workload is distributed fairly and equitably across all of the servers, each VM has been given a specific server, hence there are M servers, Equation (2), as well and each Virtual Machine has its own independent collection of resources, Equation (3).


$$\begin{array}{c} \begin{array}{c} S=\sum_{s=1}^{M}S_{s}\\ Where\text{\textbackslash{}hspace\{0.17em}\}S_{s}\text{\textbackslash{}hspace\{0.17em}\}-\text{\textbackslash{}hspace\{0.17em}\}s^{th}\text{\textbackslash{}hspace\{0.17em}\}Server \end{array} \end{array}$$ (2)

$$\begin{array}{c} \begin{array}{c} R_{vm_{\text{1}}}=\left\{R_{1,1},R_{1,2},...R_{1,j}\right\}\\ R_{vm_{\text{2}}}=\left\{R_{2,1},R2,...R_{2,j}\right\}\\ R_{vm_{n}}=\left\{R_{n,1},R_{n,2},...R_{n,j}\right\}\\ Where\text{\textbackslash{}hspace\{0.17em}\}R,\text{\textbackslash{}hspace\{0.17em}\}available\text{\textbackslash{}hspace\{0.17em}\}resources \end{array} \end{array}$$ (3)

Memory, central processing unit, disk space, and bandwidth are all considered as resources. Let us assume that there are numerous jobs in the queue requiring these resources, as represented in Equation (4).


$$\begin{array}{c} \begin{array}{c} J=\left\{J_{\text{1}},J_{\text{2}},...,J_{n}\right\}\\ Where\text{\textbackslash{}hspace\{0.17em}\}J,\text{\textbackslash{}hspace\{0.17em}\}the\text{\textbackslash{}hspace\{0.17em}\}Jobs \end{array} \end{array}$$ (4)

These jobs are loaded into the cloud server by many cloud users, say U, Equation (5).


$$\begin{array}{c} \begin{array}{c} U=\left\{U_{\text{1}},U_{\text{2}},...,U_{n}\right\}\\ Where\text{\textbackslash{}hspace\{0.17em}\;\}U,\text{\textbackslash{}hspace\{0.17em}\}Cloud\text{\textbackslash{}hspace\{0.17em}\}Users \end{array} \end{array}$$ (5)

Virtual machines (VMs) are assigned jobs in order to achieve the best possible load balancing. One server is assigned as the central server that manages incoming requests from the virtual machines (VMs) out of the system architecture’s limited number of servers. Each server is capable of processing requests, bringing the VMs up to the performance level of the most powerful server. The objective is to design an improved scheduler that assigns jobs to VMs in a manner that ensures balanced workload distribution across all machines. User characteristics will be used to maximize resource usage by using request size, number of CPU requests and number of requests sent per time period of the user. To efficiently perform cloud load balancing, it is necessary to find the hosts of adequate fitness and resources to organize the combination of VMs. The major objective is to design a scheduler that will effectively assign work to virtual machines (VMs) depending on the availability of resources.

Load balancing is optimized in terms of reaction time, cost and resource utilization in the scope of this study. These goals are to decrease the reaction time, cost minimisation, maximisation of resources and maximisation of utilisation. Load balancing is comprised of many parts. The goal of the widespread research and focused work on minimizing the reaction times, costs and resources consumption is to increase the efficiency and effectiveness. More efficient distribution and allocation of resources will increase the output. Utilizing a system’s available resources to their maximum capacity helps eliminate bottlenecks and inefficiencies. To determine the number of resources utilized, use Equation (6).


$$\begin{array}{c} \begin{array}{c} R_{utilization}=\left(\frac{R_{used}}{R_{total}}\right)\times 100\\ R_{overall-utilization}=\sum_{R=1}^{R_{resources}}\left(\frac{OU_{R_{resources}}}{R_{total}}\right)\\ Where,\text{\textbackslash{}hspace\{0.33em}\}OU_{R_{resources}}\text{\textbackslash{}hspace\{0.33em}\}=\text{\textbackslash{}hspace\{0.33em}\}Overall\text{\textbackslash{}hspace\{0.33em}\}Utilization\text{\textbackslash{}hspace\{0.33em}\}of\text{\textbackslash{}hspace\{0.33em}\}the\text{\textbackslash{}hspace\{0.33em}\}resources \end{array} \end{array}$$ (6)

By enhancing resource utilization, we can maximize the system’s capabilities and eliminate potential bottlenecks or inefficiencies. To calculate resource usage, refer to Equation (7).


$$\begin{array}{c} \begin{array}{c} T_{exe}=\overset{J}{\underset{Completion}{T}}-\overset{J}{\underset{Arrival}{T}}\text{\textbackslash{}hspace\{0.33em}\}+\text{\textbackslash{}hspace\{0.33em}\}\overset{Transmission}{\underset{delay}{T}}\\ \overset{T}{\underset{exe}{J}}=\overset{T}{\underset{start}{J}}-\overset{T}{\underset{arrival}{J}}\\ T_{response}=T_{exe}+\overset{T}{\underset{exe}{J}}\\ Where\text{\textbackslash{}hspace\{0.17em}\}T,\text{\textbackslash{}hspace\{0.17em}\}Time\text{\textbackslash{}hspace\{0.17em}\}for\text{\textbackslash{}hspace\{0.17em}\}start,execution,\text{\textbackslash{}hspace\{0.17em}\}arrival,\text{\textbackslash{}hspace\{0.17em}\}completion,delay \end{array} \end{array}$$ (7)

We observe that cost, resource utilization, and response time are interrelated when evaluating these various objectives. To achieve a well-balanced and highly efficient load-balancing system, extensive optimization and fine-tuning are required. Algorithm 1 calculates the cost of a single task by considering the resources required, the time to initiate the task, and the overall project completion time.

The execution time is intended by subtracting the start time from the finish time and dividing the result by two. The execution period is then multiplied by the total amount of resources used during the activity to determine the cost.

**Fig 1:**
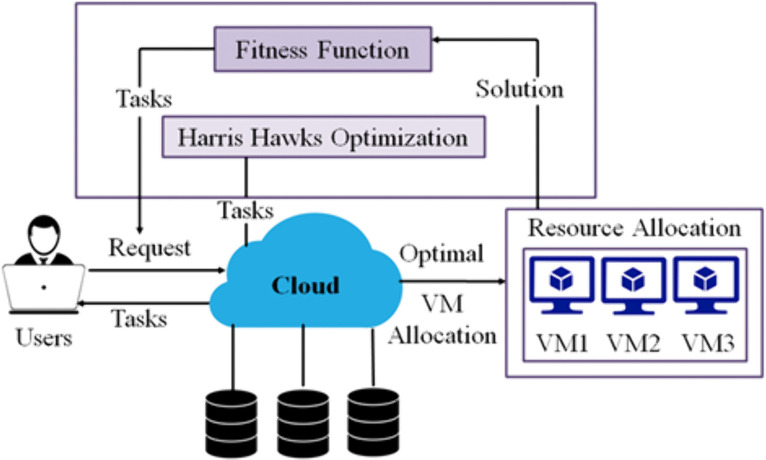
Overall architecture diagram of proposed model.

The proposed design uses the Harris Hawks Optimization (HHO) algorithm in dynamic resource allocation in cloud computing as displayed in (Fig 1). The cloud environment receives task requests made by the users in this system. These tasks are sent to the cloud controller and passed on to the Harris Hawks Optimization module where the best virtual machine (VM) allocation is done. HHO algorithm is used to measure the efficiency of different resource allocation strategies with a fitness function which is defined by parameters of task execution time, cost, and a load balance. After identifying the optimal solution, this solution is sent back to the cloud controller that proceeds to institute the optimal VM allocation among the available virtual machines (VM1, VM2, VM3). Such optimized allocation will provide a more efficient use of cloud resources, shorter response time and improved system performance. The outcomes of the performed activities are then sent back to the users. In general, the architecture combines metaheuristic intelligence and cloud infrastructure to effectively control the scheduling of tasks and provisioning of resources.

## Meta-Heuristic Harris Hawk Algorithm

One of the efficient resource allocation algorithms in cloud computing is the HHO algorithm, which is a nature-inspired algorithm that draws its inspiration on the cooperative hunting behavior of Harris hawks. This algorithm improves the functioning of the system and the simplification and optimization of its operations through sufficient allocation of resources to different activities and processes. Optimal distribution of the resources at the minimum costs and the highest degree of efficiency achievement requires several important processes.

### Initialization

Initialization of the optimization process is the first part of the Harris Hawk Optimization (HHO) algorithm. The nature of solutions that can be used is determined at this stage and initialization parameters that are to be used in the next iteration are determined.

The number of people is denoted by a letter PN and a random technique of allocating resources to each individual of the population 1 to N is calculated. To do this, the number of virtual machines (VMs), the number of CPU cores, RAM, storage space, and other allocation parameters are assigned random values. Both solutions are viable approaches of assigning resources to carry out dissimilar cloud computing jobs and are described in Algorithm 2.

### Fitness evaluation

Determine how well each population solution satisfies the requirements. Metrics such as reaction time, throughput, cost, and energy consumption are commonly used to evaluate fitness in cloud computing. A fitness function based on these performance metrics can objectively assess the effectiveness of a resource allocation method. Algorithm 3 is used to calculate the fitness value.

### Leader selection

During the leader selection phase of the HHO algorithm, the objective is to identify the solutions within the population that exhibit the highest performance, referred to as leaders. These leaders guide the search process and influence the exploration and exploitation of the solution space. Algorithm 4 illustrates the procedure used for leader selection.

The function iteratively evaluates each solution in the population, calculating its fitness value. The solution with the highest overall fitness is designated as the leader.

### Exploration

The exploration phase of the HHO algorithm explores the search space by simulating the hunting techniques of Harris Hawks, which allows finding the viable solutions to the optimization problem. During this step, a set of hawks is created, and their location as well as fitness scores are considered. Harris Hawk cooperative hunting is a model of the solution space. In this process, the solutions are updated in their positions in the population to come up with better solutions. A local search, a global search or both can be used to navigate the exploration process. This is explained in Algorithm 5.

The first positions of the hawks in the search space are generated at random to start the exploring phase. The suitability of each hawk is then assessed according to its position, which indicates its capacity to find the best answer. The hawk from the original population with the greatest fitness score is used to initialize the global best position and associated fitness value. As soon as a hawk finds a superior position in the exploration procedure, the best position world-wide is instantly updated. Each hawk in the population uses a hunting strategy to search and possibly enhance its place in every iteration of the model. In this plan, four hawks are randomly chosen in the population and identified as H1, H2, H3, and H4. The hawk that is in question at present is not included in this choice. The distance between hawks H1 and H2 is computed by a distance measure, e.g., Euclidean distance. The direction vector can be calculated as a difference between the position of H1 and the current hawk and it shows the direction, which the hawk should take. The position of the hawk is then updated according to the information. The algorithm will provide the final result (the global best position and fitness) after the exploration phase is finished. These values are a probable optimal solution of the cloud computing resource allocation optimization problem.

Algorithm 5: Exploration

Function Exploration ()

Initialize:

   P^i     // No. of Hawks in the population

   I_max     // Maximum Iteration

   m, M     // Minimum and Maximum update factor values for each individual

   P^i_pos[]   // Hawk’s position array

   F[]     // Fitness Value Array

   U_j     // Update Factor

   D_dir     // Direction of the Hawk

   B_l, B_u    // Lower and Upper Boundaries

Call PopulationInitialization()     // Algorithm 2

   P^i_pos[] ← Rand(U_l, U_u)    // Randomly Generate the position

Call FitnessEvaluation()       // Algorithm 3

   P_best ← argmin_i [F(P^i_pos[i])]  // Best Hawk in the initial population

   F_best ← min_i [F(P^i_pos[i])]  // Best Fitness in the initial population

I = 1

Repeat Until (I ≤ I_max):

   for i = 1 to P^i do:

     // Adjust the Harris Hawk movement

     Choose four Hawks (H1, H2, H3, H4) at random from P^i, excluding i

     D_collision = Euclidean_Distance(P^i_pos[H1], P^i_pos[H2])

     if F[i] > F[H1] then:

      U_j = P^i_pos[i] + (m/ D_collision)

     else:

      U_j = m - P^i_pos[i] * (M/ D_collision)

     D_direction = P_best - P^i_pos[i]

     P^i_pos[i] = P^i_pos[i] + D_direction

     // Ensure the position remains within boundaries

     P^i_pos[i] = limit(P^i_pos[i], B_l, B_u)

     F[i] = F(P^i_pos[i])// Evaluate the new position fitness

     if F[i] < F_best:

      F_best = F[i]

      P_best = P^i_pos[i]

   I = I + 1

Return (P_best, F_best)

### Computational complexity analysis

Let N denote the number of Harris hawks (search agents), D the problem dimension, and T the maximum number of iterations. The initialization stage requires O(ND) time. During each iteration, both fitness evaluation and position updating operations require O(ND)computations. Therefore, over T iterations, the overall time complexity of the proposed algorithm is O(TND). The space complexity is O(ND), corresponding to the storage of the population matrix and associated solution vectors. This confirms that the algorithm exhibits polynomial time complexity and remains computationally scalable for practical problem sizes.

## Results and discussion

### Simulation and results

The simulation results were rather helpful when evaluating the effectiveness of the proposed strategy. The study used CloudSim 4.0, a powerful simulation toolkit designed to simulate and test cloud computing environments and applications. The simulations were carried out on a Windows 10 computer equipped with an Intel Core i7 processor, 12 GB of RAM and a CPU clock frequency of 2.80 GHz.

**Fig 2:**
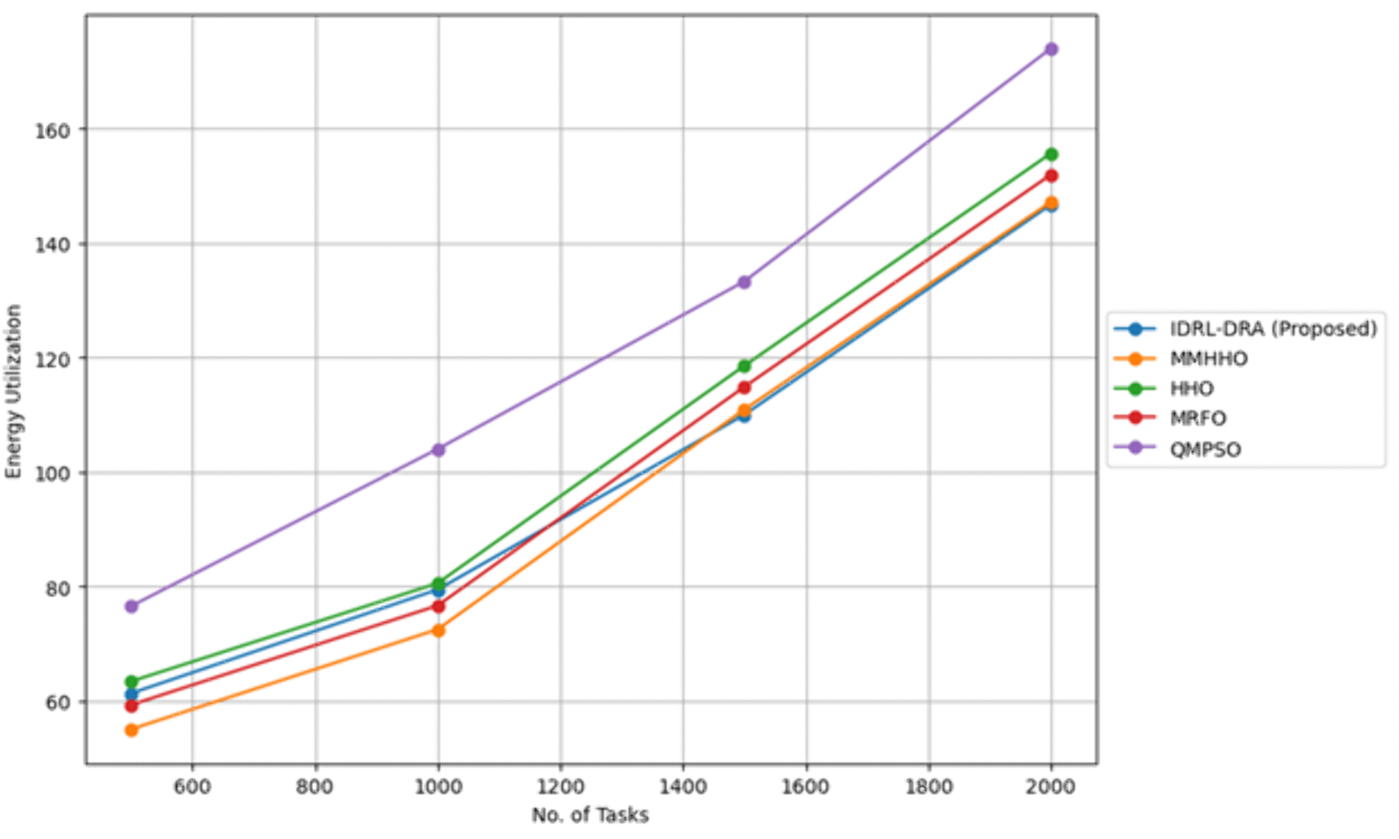
Energy utilization of dissimilar algorithms with variable number of tasks.

**Fig 3:**
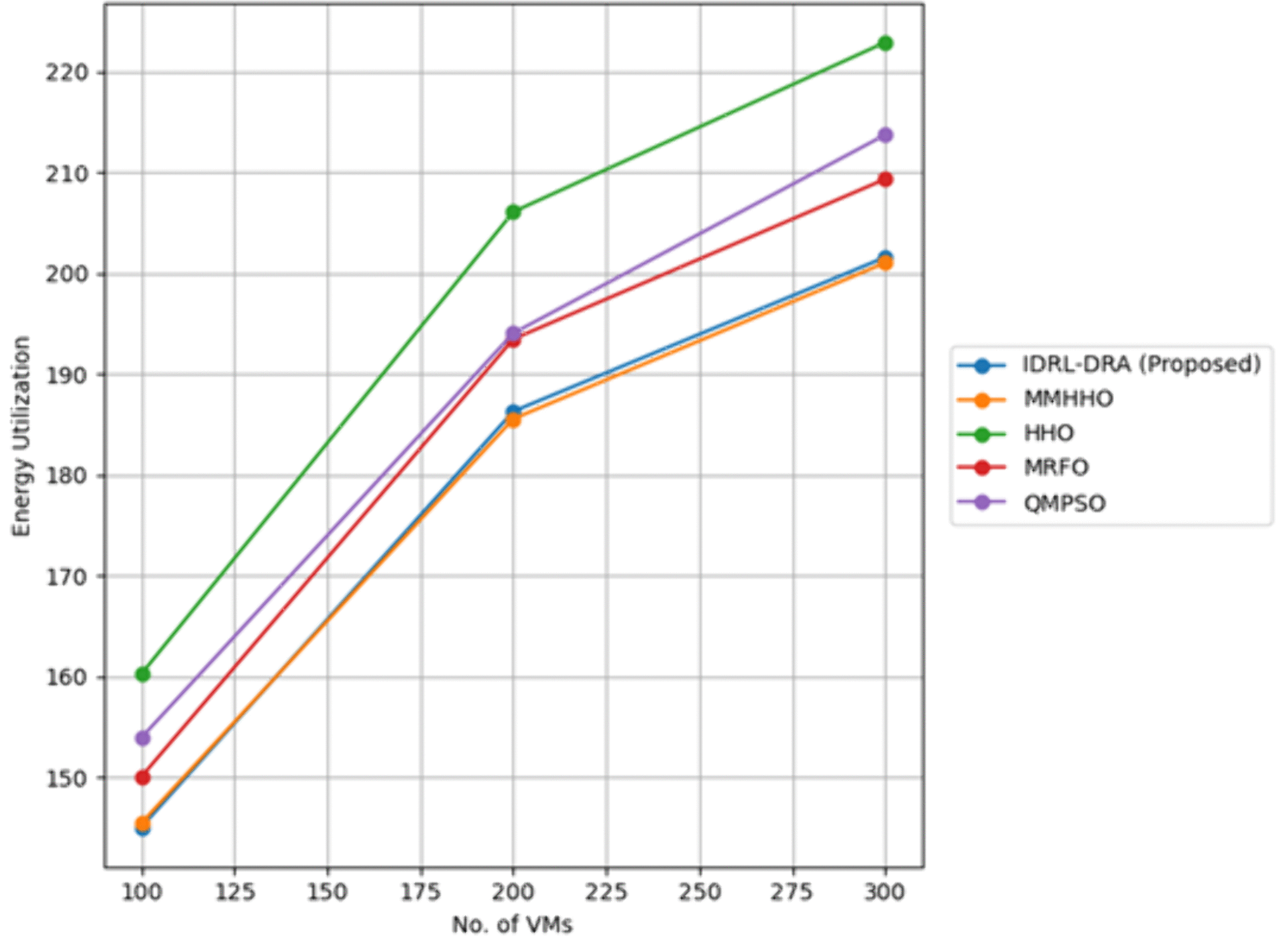
Energy utilization of different algorithms with variable number of VMs.

The efficiency of the MDLB-HHO technique has been verified through the comparison of the proposed method with four other related methods, i.e., HHO [1], MRFO [29], QMPSO [30], and MMHHO [31]. The results showed that the suggested MDLB-HHO was always better than the other approaches in several parameters and in others, they are equal to the rest. Some other criteria were also shown where the MDLB-HHO showed similar results. Figs 2 and 3 show the comparison of energy consumption of the proposed and existing methods with dissimilar numbers of jobs and the virtual machines.

**Fig 4:**
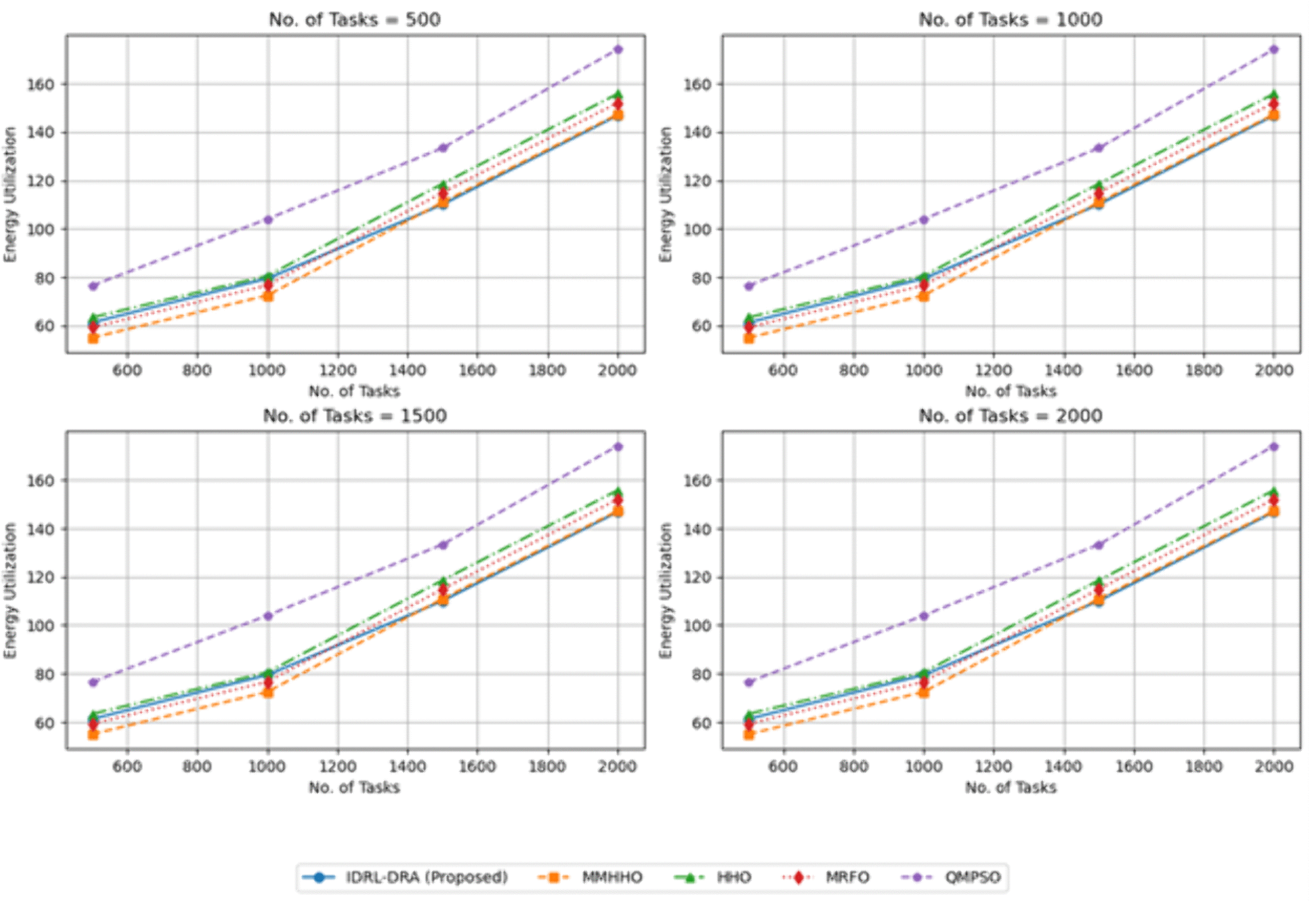
Convergence graph of energy utilization with various tasks.

**Fig 5:**
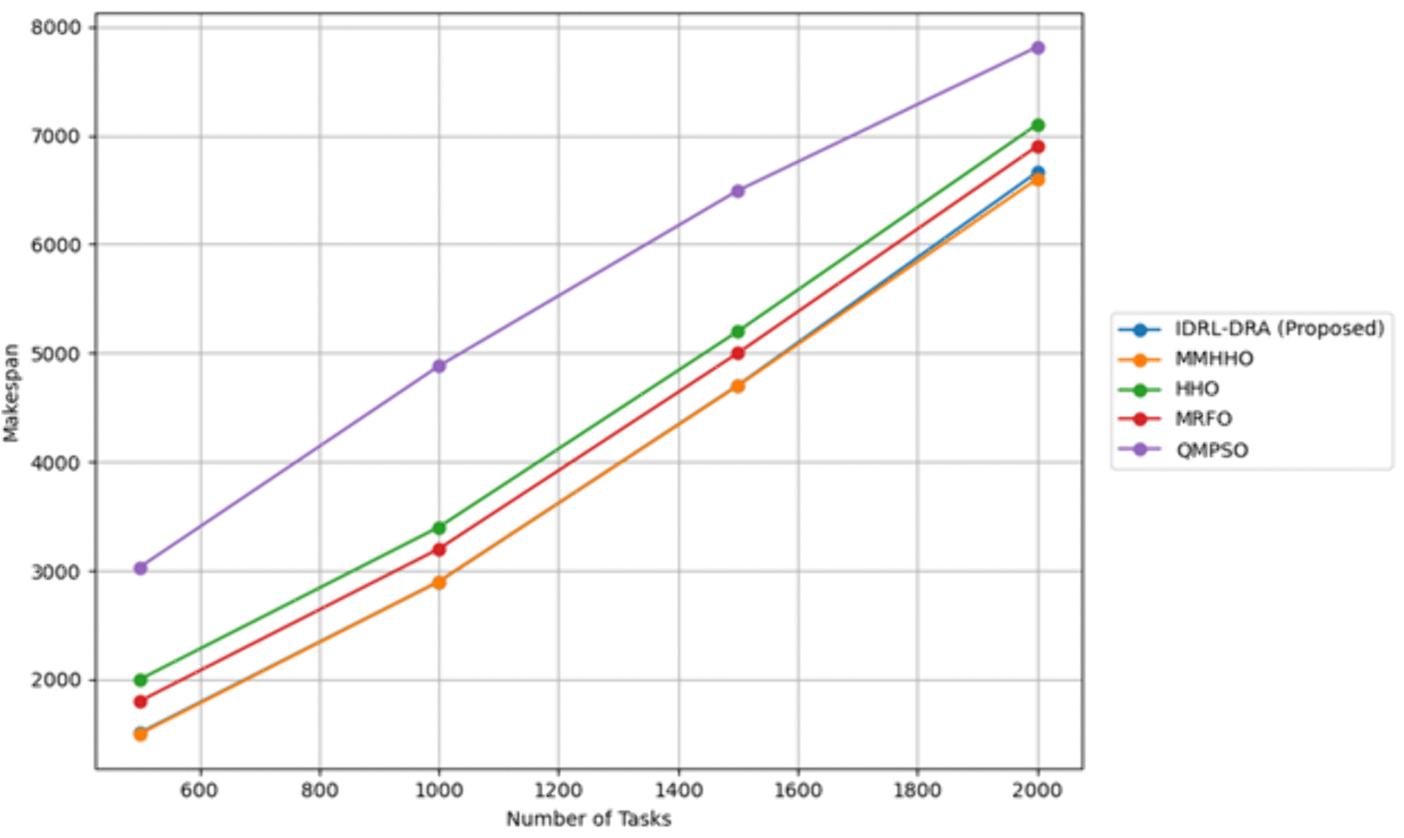
Makespan of different algorithms with variable number of tasks.

Similarly, Makespan is an important cloud computing metric that determines the time consumed to complete a chain of activities within a cloud based system. In a specific workload, it is computed as the interval of time among the beginning of the first task and the end of the last task. In cloud computing systems, decreasing Makespan is a crucial goal since it has a direct impact on the overall performance and efficiency of the system. Minimized Makespan is vital because it will result in quicker completion of tasks, better use of resources, and increased productivity in general. Reduction of Makespan enables cloud service providers to optimize the resources, maximize customer satisfaction, and become cost-effective. Scholars of cloud computing are constantly developing new algorithms of scheduling, resource management and optimization methods to minimize the execution time of cloud applications. A low Makespan in cloud systems allows them to provide quicker and more consistent services, thus improving user experience and ensuring maximum resource utilization. The Figs 4 and 5 illustrates the proposed system’s Makespan across various workloads and virtual machines.

### Discussion

The (Fig 2) shows the energy consumption of different algorithms across various task counts. The algorithms considered include MDLB-HHO (Proposed), MMHHO, HHO, MRFO, and QMPSO. Each method is evaluated for energy usage after 500, 1000, 1500, and 2000 tasks. The results reveal interesting patterns in energy consumption across the algorithms and task volumes.

The MDLB-HHO (Proposed) method consumes more energy as the workload increases: 61.29 units for 500 tasks, rising to 79.43 for 1000 tasks, 109.99 for 1500 tasks, and 146.72 for 2000 tasks. This demonstrates that energy consumption increases proportionally with workload. The MMHHO algorithm also shows a rise in energy usage with task volume, which is a characteristic of the algorithm. Its energy consumption reaches 72.47, 110.91, and 147.18 units for 1000, 1500, and 2000 tasks, respectively, after the initial 500. The HHO algorithm consistently consumes slightly more energy than both MDLB-HHO (Proposed) and MMHHO for all task levels, recording 63.38, 80.54, 118.55, and 155.69 units for 500, 1000, 1500, and 2000 tasks, respectively.

MRFO’s energy consumption is comparable to that of MDLB-HHO (Proposed), MMHHO, and HHO. For 500, 1000, 1500, and 2000 tasks, it consumes 59.27, 76.64, 114.86, and 151.97 units of energy, respectively. QMPSO, on the other hand, consistently consumes more energy across all task counts compared to the other algorithms. Specifically, for 500, 1000, 1500, and 2000 tasks, QMPSO consumes 76.54, 104, 133.3, and 174.03 units of energy, respectively, indicating higher energy usage. These values reflect a clear trend from lowest to highest energy consumption. The results show how different algorithms perform under varying workloads. MDLB-HHO (Proposed), MMHHO, HHO, and MRFO exhibit similar energy efficiency, whereas QMPSO consistently requires more energy. The recognition of such differences in energy consumption can inform the choice of algorithms depending on the task and energy efficiency.

(Fig 3) represents the energy used by the different algorithms under different numbers of virtual machines (VMs), namely, MDLB-HHO (Proposed), MMHHO, HHO, MRFO, and QMPSO. Energy consumption grows with the rise in the number of VMs in all algorithms. The trend can be explained by the fact that more processing resources are needed by more virtual machines. Both MDLB-HHO (Proposed) and MMHHO algorithms have the same trend in energy consumption, with the consumption rising between 100 and 300 VMs. Nevertheless, MMHHO always requires a bit extra energy than MDLB-HHO (Proposed) at every VM level. Both MDLB-HHO (Proposed) and MMHHO use less energy than HHO algorithm in all VM configurations, which means that HHO might require more computational resources and energy to perform tasks. The MRFO and QMPSO algorithms have fewer energy requirements compared to HHO. Nonetheless, their energy consumption also grows with the number of VMs where MRFO consumes a little bit less energy than QMPSO. These findings demonstrate how crucial energy-efficient virtual machine management is to cloud computing. Improving energy efficiency helps to lessen the carbon footprint in addition to cutting operating expenses. The convergence behavior of the suggested approach and the trends in relative energy usage are displayed in (Fig 3).

The Figs 4 and 5 displays the makespan of several methods at dissimilar task numbers. The algorithms to be tested are MDLB-HHO (Proposed), MMHHO, HHO, MRFO and QMPSO. The amount of tasks is added by 500, up to 2000. Of all the algorithms, the MDLB-HHO (Proposed) and MMHHO algorithms have the lowest makespan values in all the numbers of tasks, which shows an efficient and effective performance of the tasks. The two approaches exhibit an increasing trend in makespan with an increment in the number of tasks. Comparatively, HHO generates a little more makespan than MDLB-HHO (Proposed) and MMHHO. It is not as efficient though it does the tasks well. MRFO compares favorably with MDLB-HHO (Proposed) and MMHHO, and it is able to better address a variety of tasks than HHO, and it also has a shorter makespan. On the other hand, the QMPSO algorithm has the highest values of makespans. Its makespan grows considerably as the number of tasks grows, which is a sign of possible scalability problems. Whereas QMPSO accomplishes the tasks, it takes it longer to accomplish it. On the whole, the MDLB-HHO (Proposed) and MMHHO algorithms prove to be the most effective in time-sensitive tasks because they keep the lowest makespan values. The HHO and MRFO algorithms are also reliable though they have slightly higher values of makespan. Lastly, QMPSO is quite good at giving satisfactory results but its makespan is higher implying that there is room to improve it. These results indicate the advantages and drawbacks of each algorithm, which allows the researchers and practitioners to choose the most suitable one to balance the time of task completion and the efficiency of the operation.

**Fig 6:**
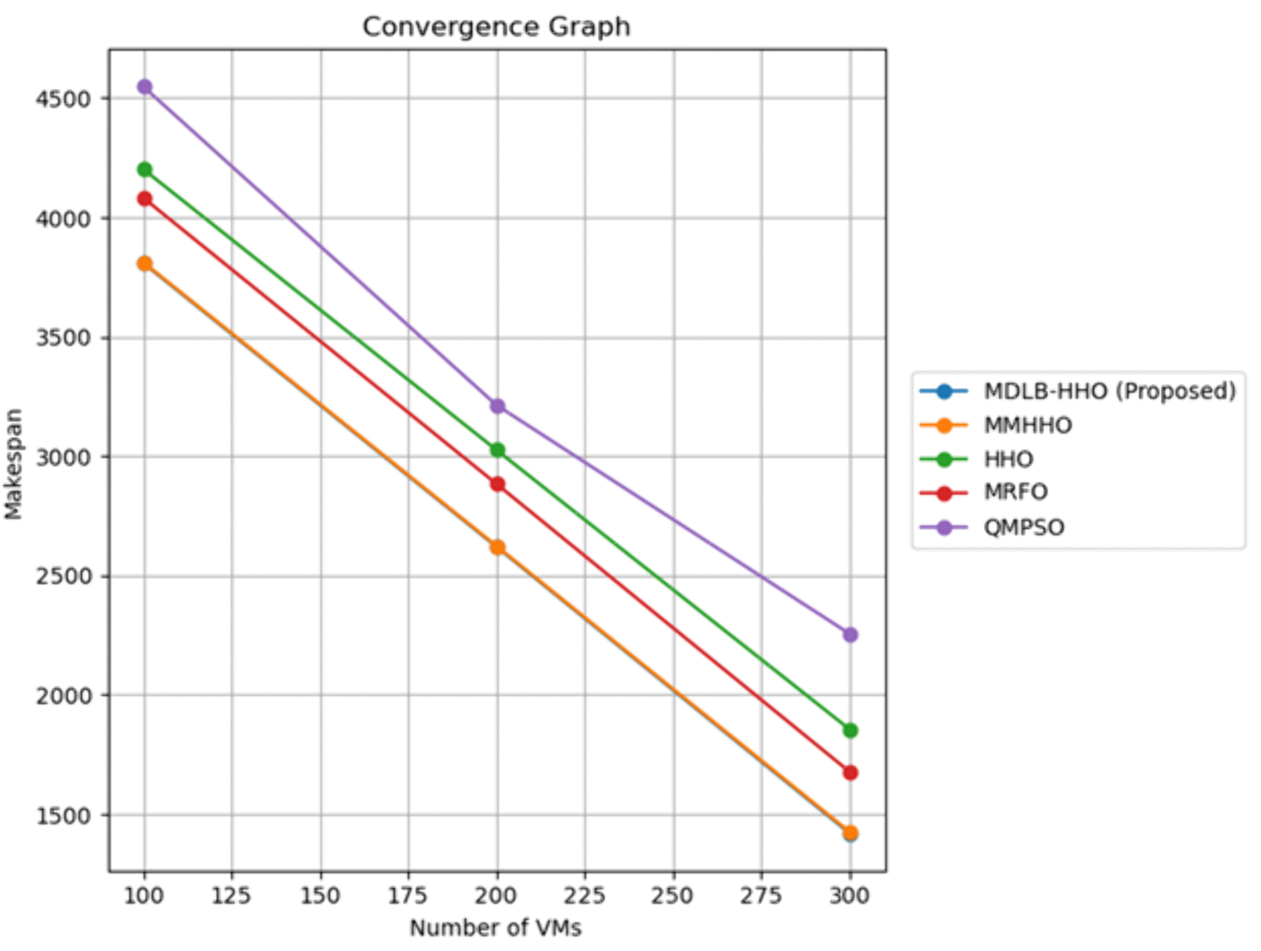
Makespan of different algorithms with variable number of VMs.

**Fig 7:**
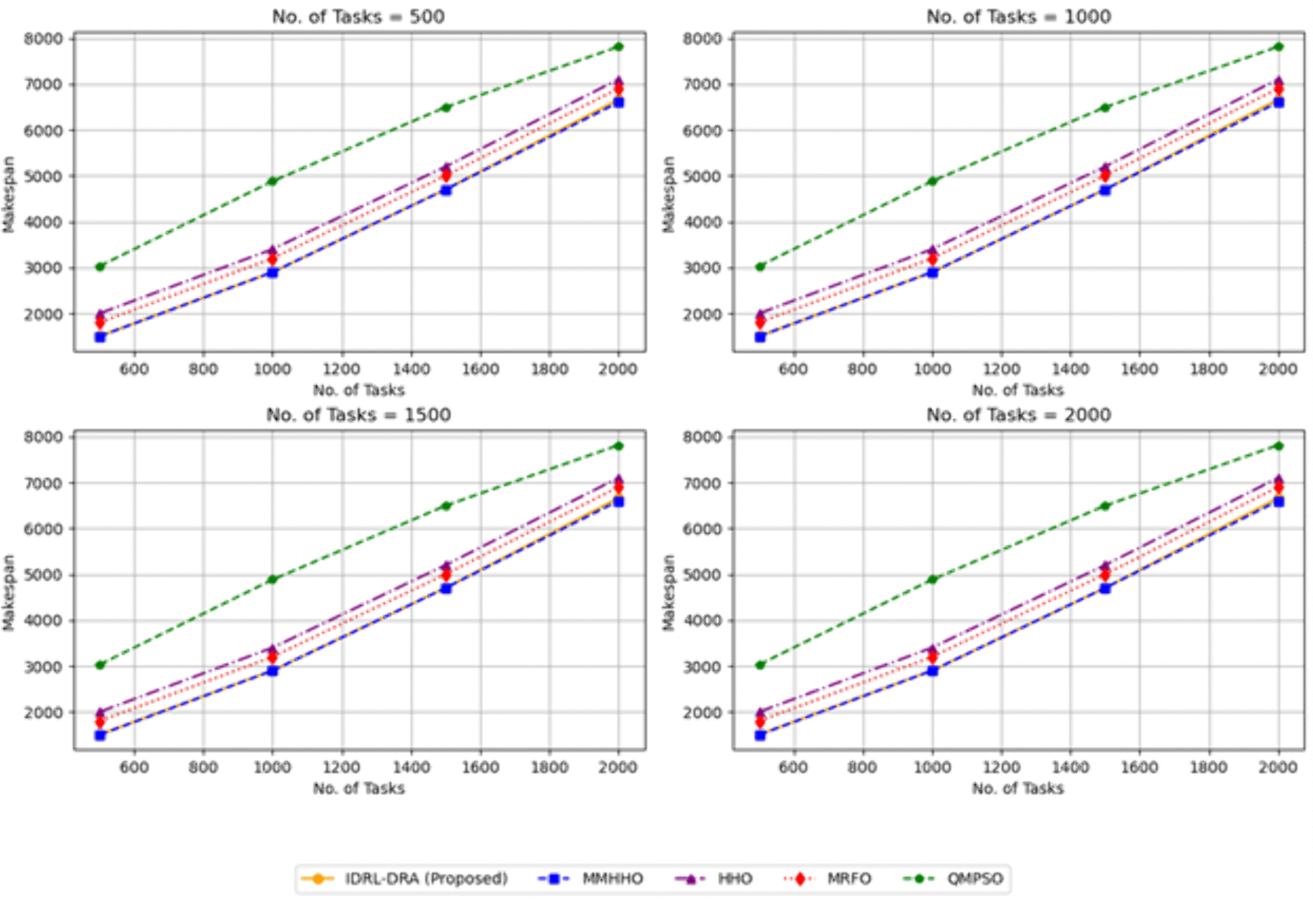
Convergence graph of makespan with various tasks.

The (Fig 6) represents makespan values of various VM counts. The analysis of data provides a number of results. MDLB-HHO (Proposed) and MMHHO have close makespan values at every VM count, which means that the two strategies are equally efficient to reduce makespan. HHO has greater makespan values than MDLB-HHO (Proposed) and MMHHO, but performs better than MRFO and QMPSO in all VM counts. Although HHO is effective, it is not efficient as MDLB-HHO (Proposed) and MMHHO. MRFO and QMPSO have the largest makespan values of all the algorithms tested, irrespective of the number of VMs, and thus they are not the best options to use in minimizing makespan in parallel computing systems. The makespan of all algorithms decreases as the number of VMs increases which suggests that the distribution of tasks among more VMs increases load balancing and efficiency. In general, data show that MDLB-HHO (Proposed) and MMHHO algorithms perform better in minimizing makespan. The convergence graph of the proposed approach with other makespan optimization algorithms is shown in (Fig 7).

#### Ablation study.

**Fig 8:**
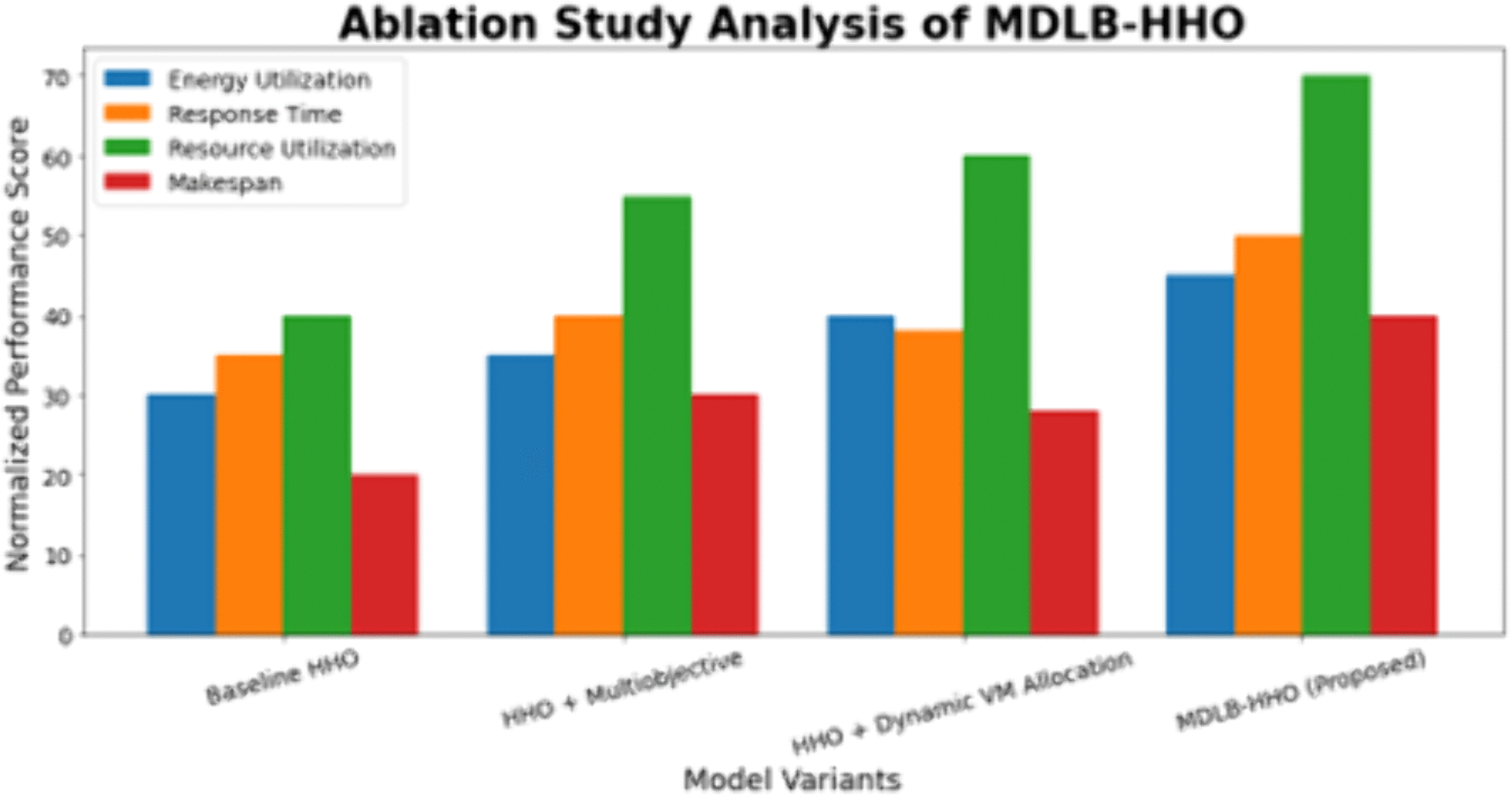
Ablation study analysis of MDLB-HHO.

To assess the role of each of the core components in the MDLB-HHO model, ablation study was performed by removing or isolating the key modules, including the multiobjective fitness function and the dynamic virtual machine (VM) allocation mechanism. The reference point was the baseline Harris Hawk Optimization (HHO) algorithm, which offered standard optimization without any task-specific trade-off management or workload adjustment. As the multiobjective function was incorporated into the baseline HHO, the significant reduction of the response time and energy efficiency was identified as a result of the balanced priorities of opposing objectives. Similarly, enabling dynamic VM allocation without the multiobjective layer enhanced resource utilization and task distribution under varying workloads. However, it lacked optimal control over cost and energy trade-offs. With the lowest energy usage, fastest response time, highest resource utilization, and shortest makespan, the complete MDLB-HHO configuration which incorporates both components performed best across all evaluation measures. This (Fig 8) illustrates how multiobjective optimization and dynamic virtual machine assignment work in concert to achieve scalable, efficient, and flexible cloud resource management (Table 2).

**Table 2:** Ablation study analysis.

Variant	HHO	Multiobjective Function	Dynamic VM Allocation	Energy Utilization	Response Time	Resource Utilization	Makespan	Result Summary
**Baseline HHO**	YES	NO	NO	Moderate	Moderate	Low	High	Standard optimization
**HHO + Multiobjective**	YES	YES	NO	Improved	Better	Moderate	Moderate	Balanced trade-offs
**HHO + Dynamic VM Allocation**	YES	NO	YES	Better	Moderate	Improved	Moderate	VM-aware optimization
**MDLB-HHO (Proposed)**	YES	YES	YES	Lowest	Fastest	Highest	Shortest	Best overall performance

#### Comparative analysis with State-of-the-art approaches.

See Table 3.

**Table 3:** Comparative analysis with State-of-the-art methods with other models.

References	Objective	Method	Optimization Goal	Performance metrics	Results
Dhabliya et al. [32]	Cloud-based dynamic load balancing	Dynamic Policies for Load Balancing	Effective load balancing.	Load balancing improvement: 12%, Response time: 8%	Improved load balancing in dynamic settings.
Dubey and Mishra [33]	Load balancing in clouds	Performance and Trust Analysis for Load Balancing	Trust-based load balancing	Trust evaluation: 16%, Performance improvement: 12%	Improved trust-based load balancing in the cloud
Singh et al. [34]	Workload distribution in fog-cloud systems.	JAYA-based Metaheuristic for the Fog-Cloud Ecosystem	Workload distribution with energy efficiency	Energy reduction: 12%, Task completion: 10%	Effective in fog-cloud systems.
Geetha et al. [35]	Optimal load balancing	Load Balancing Using Hybrid Optimization	Energy and resource optimization	Energy efficiency: 18%, Load balancing: 14%	Improved load balancing; restricted scalability.
Rostami et al. [36]	Scheduling tasks that save energy	Capuchin Search and IACO for Task Scheduling.	Energy management and task scheduling	Energy reduction: 18%, Task completion: 12%	Improved work scheduling and energy efficiency.
Kumar and Karri [37]	Cost-aware task scheduling	AGWO Hybrid: Task Scheduling	Cost and task scheduling optimization	Cost reduction: 14%, Task allocation: 9%	Efficient scheduling in cloud fog systems.
**Our Proposed Model**	**Cloud computing uses dynamic load balancing and resource allocation.**	**MDLB-HHO (Harris Hawk Optimization)**	**Optimize response time, resource utilization, energy efficiency, and cost**	**Response time:18%, Resource utilization: 16%, Energy efficiency: 14%, Cost: 12%**	**Enhanced efficiency and scalability in dynamic cloud settings**

## Conclusions

The MDLB-HHO algorithm is used in this article to introduce an evolutionary method for resource allocation and dynamic load balancing in cloud computing. The suggested method is a multiobjective meta-heuristic strategy that seeks to minimize Makespan time and maximize resource consumption. Being a multiobjective optimization problem, load balancing issue is associated with the allocation of jobs to virtual machines (VMs) on the fly regarding resource consumption and work distribution in real-time. MDLB-HHO algorithm is very effective in search and discovery of the best solution to the problem using the adaptive position-updating techniques and iteration of the Harris Hawks and search in the solution space. The solution also has several objectives that it achieves through the incorporation of the collaborative hunting approach of hawks, which renders it suitable in dynamic cloud environments. The proposed multiobjective optimization scheme integrates Makespan time, cost and resource consumption to significantly improve effectiveness and performance of cloud computing systems. This project ends with the development of a better load balancing and resource allocation technique founded on the Harris Hawk Optimization (HHO) algorithm. Experimental data indicate that the proposed MDLB-HHO approach is more effective than the existing methods in reducing Makespan and maximizing resource utilization. The approach ensures cost-efficient and efficient cloud systems load balancing by addressing the problem as a multiobjective optimization task. The findings also indicate that there is room of future research particularly in the field of hybrid optimization strategies to improve the efficiency of systems in cloud computing.
