# Supplementary material for: Multiobjective dynamic resource allocation in cloud computing using Harris Hawk Optimization Algorithm (MDLB-HHO)
Source: PLoS One. 2026 Jun 25;21(6):e0351653. doi: 10.1371/journal.pone.0351653 (PMC13298939; doi:10.1371/journal.pone.0351653)
Supplement: Attachment — Submitted filename: Response to Reviewers.docx [file pone.0351653.s002.docx]

**Dear Dinesh Kumar Nishad,**

Thank you for the opportunity to revise our manuscript, " Multiobjective dynamic resource allocation in cloud computing using Harris Hawk optimization algorithm (MDLB-HHO)" (Manuscript ID: PONE-D-25-38807). We are grateful to you and the reviewers for the constructive feedback and the time invested in evaluating our work.

We have carefully considered each point raised. The reviewers' insights have been invaluable, allowing us to clarify our methodology and strengthen the impact of our findings. In the revised version, we have addressed all concerns.

Below, we provide a point-by-point response to the reviewers’ comments. All modifications in the manuscript have been highlighted in yellow for your convenience. We believe these revisions have significantly enhanced the clarity and rigor of our study and hope it is now suitable for publication in PLOS ONE.

| **Comment** | **Response** |
| --- | --- |
| **Academic Editor Comments** | |
| Please ensure that you refer to Figures 4 and 8 in your text as, if accepted, production will need this reference to link the reader to the figures. | There are 6 figures and all the figures are cited in the text in appropriately. |
| We note you have included a table to which you do not refer in the text of your manuscript. Please ensure that you refer to Tables 1, 2, and 3 in your text; if accepted, production will need this reference to link the reader to the Tables. | All the tables are properly numbered, referenced and cited in the relevant places in the manuscript. |
| **Reviewer #1:** | |
| 1. What are the motivations of researchers to consider Harris Hawks optimization besides many Reinforcement learning approaches? | The motivation for selecting the Harris Hawks Optimization (HHO) algorithm instead of Reinforcement Learning (RL) approaches is mainly in the nature of the cloud load balancing problem and adaptability requirements.  The selection of Harris Hawks Optimization (HHO) is mainly motivated by:  Its natural suitability for multiobjective optimization.  Lower computational overhead compared to RL.  No training or reward modeling requirement.  Strong exploration–exploitation balance.  Scalability to high-dimensional cloud environments.  Stable and reproducible convergence behavior.  Addressing a research gap in cloud-based HHO applications. |
| 2. What are your inferences from the existing literature? | A careful survey of the existing literature indicates that cloud load balancing is fundamentally a multiobjective problem requiring the consideration of response time, makespan, energy consumption, cost, and resource utilization. While meta-heuristic algorithms such as GA, PSO, ACO, WOA, and GWO dominate current research due to their global search capabilities, most studies either focus on incremental modifications or hybridizations of established techniques rather than exploring structurally different optimizers in detail. Furthermore, although energy-efficient and green cloud computing have gained increasing attention, many existing approaches improve one metric at the expense of others, resulting in suboptimal trade-off management. The literature also reveals limited comprehensive investigation of Harris Hawks Optimization (HHO) in dynamic, multiobjective cloud scheduling environments, despite its strong exploration–exploitation balance and convergence properties demonstrated in other optimization domains. Additionally, adaptability under highly dynamic workloads and scalability in large heterogeneous cloud infrastructures remain insufficiently addressed. These collective observations highlight a clear research gap and strongly motivated the development of the proposed multiobjective HHO-based dynamic load balancing framework. |
| 3. Mathematical modeling is weak in the manuscript. | We sincerely thank the Reviewer for this valuable observation regarding the strength of the mathematical modeling in the manuscript. We respectfully clarify that while the paper focused more on algorithmic design and experimental validation, the underlying mathematical framework was present but not elaborated with sufficient depth and clarity. |
| 4. Algorithm is not rigorously explained. | We respectfully clarify that the proposed algorithm itself is already grounded in the standard and well-established mathematical framework of Harris Hawks Optimization (HHO). Our primary contribution lies in the problem modeling, multiobjective fitness formulation, and application-specific adaptation rather than in redesigning or modifying the underlying HHO update equations. Since the algorithmic steps strictly follow the canonical HHO structure with clearly defined mathematical expressions and solution update rules, we believe that the methodological foundation is inherently rigorous. |
| 5. Complexity of the algorithm haven't identified but they have done the ablation study. | We sincerely thank the reviewer for highlighting the importance of analyzing the computational complexity of the proposed algorithm. While the ablation study was conducted to evaluate the contribution and effectiveness of individual components of the framework, we agree that a formal complexity analysis is essential to assess its computational efficiency and scalability.  Section 5.5 Computational Complexity Analysis is added in the revised manuscript. |
| 6. Many of the authors have already addressed these metrics what is your thought on this? | Our intention is not merely to reuse established metrics, but to employ them in a unified and application-specific framework to ensure fair comparability with existing approaches. In our work, these metrics are systematically integrated within the proposed optimization framework to highlight performance of the proposed study. |
| 7. Is your approach is working well with these given settings or it can be adaptable to any type of workload. Please justify. If so, please give us the reasons. | The proposed approach is not restricted to the specific experimental settings considered in the manuscript. Since the algorithm is population-based and driven by a fitness function formulation, it can naturally adapt to different workload patterns. Therefore, as long as workload characteristics can be quantitatively modeled within the fitness function, the approach remains adaptable to varying workload environments. |
| **Reviewer #2:** | |
| The paper tackles an interesting, highly relevant, and complex issue: multi-objective dynamic load balancing in cloud computing, using a meta-heuristic approach called MDLB-HHO. It shows promising simulation results that outperform existing methods. The literature review clearly identifies the gap in applying HHO to this specific problem. | We sincerely thank the Reviewer for the encouraging and constructive feedback. It is particularly gratifying that the Reviewer acknowledges the significance of applying the MDLB-HHO framework to this domain and recognizes the clarity of the identified research gap in the literature. This positive assessment reinforces our motivation to further refine and strengthen the manuscript, and we remain committed to improving its clarity and technical depth in line with the reviewers’ valuable suggestions. |
| However, concerns about the practical feasibility and cost of the solution are significant and cannot be ignored. Especially for this journal, the authors must provide a detailed Computational Complexity Analysis (including both time and space complexity) of the MDLB-HHO algorithm and discuss its implications for a real-time system. They should also compare its computational cost to that of other algorithms. | In response to this valuable suggestion, we have incorporated a new subsection titled **“5.5 Computational Complexity Analysis”** in the revised manuscript. This section presents a detailed evaluation of both the time and space complexity of the proposed MDLB-HHO algorithm, along with a discussion on its suitability for real-time cloud environments. |
| Additionally, the authors should include a discussion on the practical challenges of deployment and the limitations of relying solely on simulations, outlining future work to test the algorithm on areal or emulated cloud infrastructure with production-level traffic characteristics. The paper should be revised to clearly explain the novelty of the multiobjective fitness function, its parameters, and how it effectively balances the conflicting objectives mentioned (e.g., resource utilization versus response time). | Section **6. Results and Discussion** has been expanded to explicitly address the practical deployment challenges, the limitations of simulation-based evaluation, and the need for validation on real or emulated cloud infrastructures with production-level workloads. |
